# Rapid detection and diagnosis of herpetic keratitis using quantitative microfluidic polymerase chain reaction system for herpes simplex and varicella-zoster virus DNA: a case series

**DOI:** 10.1186/s12886-023-02938-w

**Published:** 2023-04-25

**Authors:** Akira Hirota, Jun Shoji, Noriko Inada, Rumi Adachi, Yukiko Tonozuka, Satoru Yamagami

**Affiliations:** Division of Ophthalmology, Department of Visual Sciences, Nihon University School of Medicine, 30-1 Oyaguchi-Kamichou, Itabashi-Ku, Tokyo, 173-8610 Japan

**Keywords:** Herpes simplex virus, Varicella-zoster virus, Quantitative polymerase chain reaction, Keratitis, Conjunctivitis

## Abstract

**Background:**

A microfluidic real-time polymerase chain reaction (PCR) system can rapidly detect the viral DNA in specimens. Detection of herpes simplex virus (HSV) and varicella-zoster virus (VZV) DNA in tears is a useful diagnostic tool for herpes simplex virus keratitis (HSK) and herpes zoster ophthalmicus (HZO).

**Methods:**

In total, 20 patients were included in this cross-sectional study. Among them, 8 patients with infectious epithelial HSK and 12 patients with HZO were included in HSK and HZO groups, respectively. In addition, 8 patients with non-herpetic keratitis and 4 healthy individuals without keratitis were included in the control group. Numbers of HSV and VZV DNA copies in tears of all patients and individuals were evaluated using a microfluidic real-time PCR system. Regarding HSV/VZV DNA test, tear specimens were collected by filter paper method using Schirmer’s test paper, and subsequently, DNA was extracted from the filter paper using an automated nucleic acid extractor. Afterward, quantitative PCR was performed using a microfluidic real-time PCR system.

**Results:**

From tear collection to real-time PCR result determination, the HSV/VZV DNA test took approximately 40 min. In the HSK group, the sensitivity and specificity of the HSV DNA tests were 100% each. The median value (range) of number of HSV DNA copies for affected eyes was 3.4 × 10^5^ copies/μL (under a lower detection limit of 7.6). In the HZO group, the sensitivity and specificity of the VZV DNA tests were 100% each. The median value (range) of number of VZV DNA copies for affected eyes was 5.3 × 10^5^ copies/μL (under a lower detection limit of 5.6 × 10^–2^).

**Conclusion:**

In conclusion, quantitative PCR for HSV and VZV DNA in tears using a microfluidic real-time PCR system is useful for diagnosing and monitoring HSK and HZO.

## Background

Herpes simplex keratitis (HSK) is a keratitis caused by primary infection or reactivation of herpes simplex virus (HSV), with a wide variety of clinical manifestations including infectious epithelial keratitis, neurotrophic keratitis, stromal keratitis, and endotheliitis [1]. Typical infectious epithelial keratitis, including dendritic keratitis, and geographic keratitis is diagnosed by clinical findings and viral culture methods. However, atypical infectious epithelial keratitis, neurotrophic keratitis, stromal keratitis, and endotheliitis are often difficult to diagnose clinically. Consequently, supporting tests such as antigen testing and nucleic acid testing using polymerase chain reaction (PCR) methods have been considered [2–4]. According to a previous study, quantitative PCR is a useful diagnostic tool for infectious epithelial keratitis caused by HSV [5, 6].

Herpes zoster, caused by reactivation of the latent varicella-zoster virus (VZV) within a unilateral first branch of the trigeminal nerve, is complicated by herpes zoster ophthalmicus (HZO). HZO is characterized by the development of a variety of anterior eye diseases, including blepharitis, conjunctivitis, keratitis, and episcleritis [7]. Diagnosis of herpes zoster is based on clinical findings of skin rash, results of morphologic test by Tzanck smear [8], or detection of VZV DNA by PCR in the specimens obtained from the skin rash of patients with acute herpes zoster [9]. In clinical practice, refractory keratitis and episcleritis lasting for weeks to months are observed in some patients with HZO. Therefore, an ophthalmologic test to assess the viral activity of VZV in these patients is long-awaited. Quantitative evaluation of the VZV DNA using real-time PCR has been reported to be useful in determining the severity of the disease and the response to treatment [10].

However, the conventional real-time PCR method for estimating the amount of HSV and VZV DNA has been deemed unsuitable to use as a clinical test because of the complicated procedure and the time required for measurement.

This study aimed to establish a rapid detection method for measuring the amount of HSV/VZV DNA in tear fluid using a microfluidic real-time PCR system and to investigate its usefulness in the diagnosis of HSV and VZV keratitis.

## Methods

This cross-sectional and observational study was approved by the Institutional Review Board of the Nihon University Itabashi hospital (approval number: RK-190709–2) and adhered to the tenets of the Declaration of Helsinki. Written informed consent was obtained from all patients enrolled in this study.

### Patients

In total, 20 patients with clinically diagnosed HSK and HZO, who visited Nihon University Itabashi Hospital, Tokyo, Japan between September 2019 and June 2022 were enrolled in this study. In addition, 8 patients with non-herpetic keratitis and 4 healthy individuals without keratitis were enrolled as controls. Next, the patients and controls were divided into the following three groups: HSK (*n* = 8), HZO (*n* = 12), and control (*n* = 12). The patients with non-herpetic keratitis included the following: two with atopic keratoconjunctivitis; two with phlyctenular keratoconjunctivitis; two with allergic conjunctivitis; one with Stevens-Johnson syndrome; and one with ocular cicatricial pemphigoid. Demographic data are presented in Tables 1, 2, and 3.

**Table 1:** List of the patients with HSV keratitis (HSK group)

Case	Sex	Age	Affected eye	Clinical stage	Clinical form	AHA	AD	HSV DNA (copies/μL)	
								**Right eye**	**Left eye**
HSV1	F	77	R	Active	Dendritic keratitis	+	–	4.6 × 10^7^	ND
HSV2	M	45	R	Active	Geographic keratitis	+	+	2.3 × 10^7^	ND
HSV3	M	44	R	Active	Dendritic keratitis	+	+	8.3 × 10^6^	NT
HSV4	M	38	R	Active	Geographic keratitis	–	+	4.6 × 10^6^	ND
HSV5	M	45	R	Active	Geographic keratitis	–	+	2.2 × 10^6^	ND
HSV6	M	55	L	Active	Dendritic keratitis	–	+	ND	2.8 × 10^5^
HSV7	F	82	R	Active	Recurrent dendritic keratitis after interstitial keratitis	+	–	1.3 × 10^5^	NT
HSV8	F	56	R	Active	Dendritic keratitis	+	+	3.5 × 10^4^	ND

Abbreviations: *M* male, *F* female, *R* right eye, *L* left eye, *AHA* anti-herpes virus agent, *AD* atopic dermatitis, *ND* not detected, *NT* not tested, *HSV* herpes simplex virus

**Table 2:** List of the patients with VZV keratitis (HZO group)

Case	Sex	Age	Affected eye	Clinical stage	Clinical form	AHA	VZV DNA (copies/μL)	
							**Right eye**	**Left eye**
VZV1	M	70	R	Active	Blepharitis, scleritis,keratoconjunctivitis	+	4.0 × 10^8^	6.7 × 10^3^
VZV2	M	49	R	Active	Blepharitis, scleritis,keratoconjunctivitis	+	9.4 × 10^7^	7.7 × 10^1^
VZV3	M	47	R	Active	Blepharitis, scleritis,keratoconjunctivitis	+	3.5 × 10^7^	9.9 × 10^3^
VZV4	M	79	R	Active	Blepharitis, scleritis,conjunctivitis	–	4.6 × 10^6^	3.2 × 10^3^
VZV5	F	74	R	Active	Blepharitis,keratoconjunctivitis	+	3.9 × 10^6^	2.1 × 10^2^
VZV6	M	82	R	Active	Blepharitis,keratoconjunctivitis	–	8.6 × 10^5^	2.8 × 10^4^
VZV7	F	27	R	Active	Blepharitis,conjunctivitis	+	1.9 × 10^5^	1.2 × 10^2^
VZV8	F	47	L	Active	Blepharitis,conjunctivitis	+	ND	1.6 × 10^5^
VZV9	F	77	R	Active	Keratoconjunctivitis	–	2.6 × 10^4^	ND
VZV10	F	89	L	Active	Blepharitis,conjunctivitis	–	2.3 × 10^4^	6.7 × 10^3^
VZV11	F	73	L	Active	Blepharitis,conjunctivitis	–	NT	4.6 × 10^3^
VZV12	M	22	L	Active	Keratoconjunctivitis	+	ND	3.2 × 10^3^

Abbreviations: *M* male, *F* female, *R* right eye, *L* left eye, *AHA* anti-herpes virus agent, *ND* not detected, *NT* not tested, *VZV* varicella-zoster virus

**Table 3:** List of the patients with non-herpetic keratitis and healthy individual without keratitis (Control group)

	**Total**	**Patients with non-herpetic keratitis**	**Healthy individuals without keratitis**
No. of patients	12	8	4
Age, years (mean ± SD)	36.9 ± 13.3	38.8 ± 16.0	33.3 ± 1.30
Sex ratio (Male: Female)	2:1	1:1	Man only
Keratitis and/or conjunctivitis		AC: 2 cases AKC: 2 cases PKC: 2 cases SJS: 1 case OCP: 1 case	Non
Negative ratio	12/12 (100%)	8/8 (100%)	4/4 (100%)
Positive ratio	0/12 (0%)	0/8 (0%)	0/4 (0%)

Abbreviations: *SD* standard deviation, *AC* allergic conjunctivitis, *AKC* atopic keratoconjunctivitis, *PKC* phlyctenular keratoconjunctivitis, *SJS* Stevens-Johnson syndrome, *OCP*, ocular cicatricial pemphigoid

The inclusion criteria for the HSK group were patients who experienced infectious epithelial keratitis—diagnosed based on the clinical findings of dendritic or geographic keratitis by slit-lamp examination. Exclusion criteria were the following: 1) patients who had stromal keratitis or endotheliitis form of HSK; and 2) patients who had atypical infectious epithelial keratitis.

The inclusion criteria for the HZO group were patients who experienced VZV keratitis, conjunctivitis, and/or scleritis associated with varicella-zoster dermatitis in the area of distribution of the first branch of the trigeminal nerve. In addition, the exclusion criteria were the following: 1) patients experiencing uveitis without keratitis, conjunctivitis, and scleritis and 2) patients with varicella (initial infection).

### Real-time PCR for HSV and VZV DNA

#### Tear sampling method

The tear specimens were collected by filter paper method using Schirmer’s test paper (Schirmer Tear Production Measuring Strips, Ayumi Pharmaceutical Co., Tokyo, Japan) [5]. According to the Schirmer test technique, the Schirmer’s test paper was inserted into the lower conjunctival fornix and left in place for 1 min. Next, a 5 mm tip of the Schirmer’s test paper was cut off to extract the DNA. Tear specimens collected using Schirmer’s test paper were dissolved in cold lysis buffer [0.32 M Sucrose 5.48 g, 10 mM Trig/HCL 0.5 mL, 5 mM MgCl_2_ 0.25 mL, and 1% Tween 20 (polyoxyethylene sorbitan monolaurate) 0.5 mL], and DNA was extracted using automated nucleic acid extractor (mag LEAD® , Hitachi, Tokyo, Japan), which required approximately 26 min.

#### Microfluidic real-time PCR system for HSV and VZV DNA

### Measurement of tear specimens

Quantitative real-time PCR was performed using a microfluidic real-time PCR system (GeneSoC®, Kyorin, Tokyo, Japan). Gene SoC® can show three colors; therefore, simultaneous measurements of HSV DNA (Cy5) and VZV DNA (FAM) were performed in this study. Since the time required for real-time PCR was approximately 12 min, the HSV and VZV DNA test results were available in 40 min following the collection of tears.

The PCR mix contained cold lysis buffer including DNA extracts 200 μL, HSV-1 primer & probe [Probe (Cy5): TAG TGG GCC TCC ATG GG, HSV1F: GGG CCG TGA TTT TGT TTG TC, HSV1R: CCG CCA AGG CAT ATT TGC] 2.5 μL, HHV3 TaqMan probe (FAM) (TaqMan gene expression assay, Life Technologies Japan, Carlsbad, Tokyo, Japan) 1.0 μL, 10 × FAST Buffer I (Takara Bio Inc, Shiga, Japan) 2.0 μL, dNTP Mixture (Takara Bio) 1.6 μL, SpeedSTAR™ HS DNA Polymerase (Takara Bio) 0.4 μL, and distilled water 3.9 μL.

### Preparation of calibration curve

For the preparation of the calibration curve, commercially available genomic DNA of Human herpes virus 1 (HHV-1, ATCC® VR-589DQ™) (American Type Culture Collection, VA, USA) 7.6 × 10^5^ copies/μL was prepared as a standard solution for HSV DNA, and genomic DNA of Human herpes virus 3 (HHV-3, ATCC® VR-1367DQ™) (American Type Culture Collection) 5.6 × 10^5^ copies/μL was prepared as a standard solution for VZV DNA. A dilution series of each standard viral DNA solution was prepared and measured three times each using the microfluidic real-time PCR system. Then, the mean value of the measured values was calculated to create a calibration curve.

## Results

### Preparation of calibration curve in quantitative PCR for HSV and VZV DNA

**Fig. 1:**
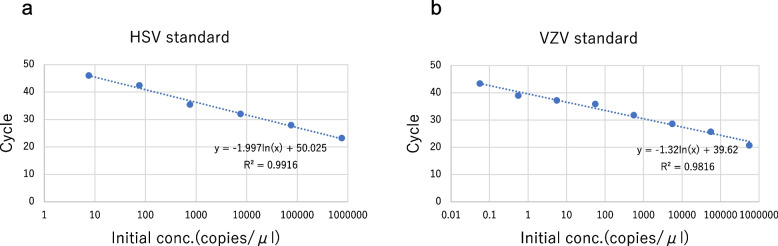
Calibration curve in quantitative PCR for HSV and VZV DNA in tears. We developed a calibration curve to determine the amount of HSV and VZV DNA in tears. **a**. The measurement ranges for HSV DNA tests in tears were 7.6 × 10^1^–7.6 × 10^5^ copies/μL. **b**. The measurement ranges for VZV DNA tests in tears were 5.6 × 10^–3^–5.6 × 10^5^ copies/μL. PCR, polymerase chain reaction; HSV, herpes simplex virus; VZV, varicella-zoster virus

We developed a calibration curve to determine the amount of HSV and VZV DNA in tears (Fig. 1). The measurement ranges for HSV DNA and VZV DNA tests in tears were 7.6 × 10^1^–7.6 × 10^5^ and 5.6 × 10^–3^–5.6 × 10^5^ copies/μL, respectively.

### Analysis of sensitivity, specificity, positive predictive value, and negative predictive value

Tear specimens with a measured value were classified as positive, and those with a value under the detection limit were classified as negative (Table 4). In the HSK group, all eight patients had positive results of the HSV DNA test (in tears). In the HZO group, all 12 patients had positive results of the VZV DNA test (in tears). Furthermore, all 12 individuals in the control group had negative results for both HSV and VZV DNA tests.

**Table 4:** Sensitivity, specificity, PPV, and NPV in quantitative polymerase chain reaction for detecting HSV and VZV DNA

		**HSV DNA**		**VZV DNA**	
		Positive	Negative	Positive	Negative
**Ocular surface diseases**	Yes	8	0	12	0
	No	0	12	0	12
		Sensitivity	100%	Sensitivity	100%
		Specificity	100%	Specificity	100%
		PPV	100%	PPV	100%
		NPV	100%	NPV	100%

Abbreviations: *HSV* herpes simplex virus, *VZV* varicella-zoster virus, *PPV* positive predictive value, *NPV* negative predictive value

The sensitivity, specificity, positive predictive value, and negative predictive value of both HSV and VZV DNA tests were 100% each (Table 4). In addition, no HSK and HZO patients had positive VZV and HSV DNA tests, respectively (Table 5).

**Table 5:** Results of simultaneous measurement of HSV DNA and VZV DNA

		**VZV DNA (HZO group)**	
		**Positive**	**Negative**
**HSV DNA (HSK group)**	Positive	0	8
	Negative	12	0

Abbreviations: *HSV* herpes simplex virus, *VZV* varicella-zoster virus

### HSV and VZV DNA levels in tears of patients

HSV and VZV DNA levels in the tears of each patient are shown in Tables 1 and 2, respectively. HSV and VZV DNA levels of all patients were within the measurement range. Median values of HSV and VZV DNA levels in tears for affected eyes were 3.4 × 10^5^ and 5.3 × 10^5^ copies/μL, respectively.

**Fig. 2:**
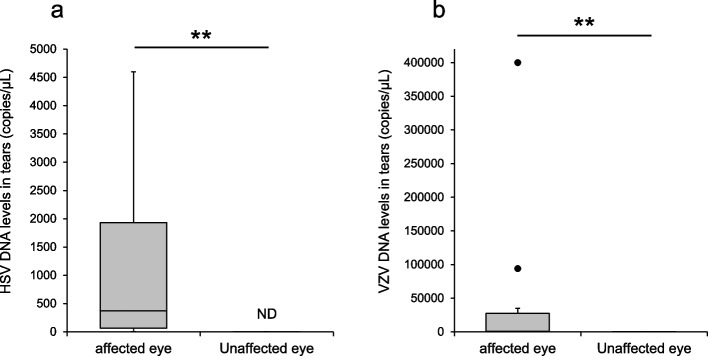
Comparison of HSV and VZV DNA levels in tears between affected and unaffected eyes. **a**. HSV DNA levels in tears of affected eyes are significantly higher than those of unaffected eyes. HSV DNA levels in all tear samples in unaffected eyes are under the detection limit. **b**. VZV DNA levels in tears of affected eyes are significantly higher than those of unaffected eyes. **, *p* < 0.01, Mann–Whitney U-test; HSV, herpes simplex virus; VZV, varicella-zoster virus; ND, not detected

In the HSV group, all affected eyes were positive, while all unaffected eyes were under the detection limit of the HSV DNA tests (Fig. 2a). Six of the eight patients were complicated by atopic dermatitis with atopic keratoconjunctivitis (AKC). All six patients with AKC were treated with anti-allergic eye drops, steroid eye drops, and immunosuppressive eye drops, and none were on immunotherapy.

In the HZO group, all eyes (affected and unaffected) tested positive in VZV DNA tests, although the VZV DNA levels in tears in the affected eyes were significantly higher than in the unaffected ones (Fig. 2b).

### Representative case of HSV keratitis

**Fig. 3:**
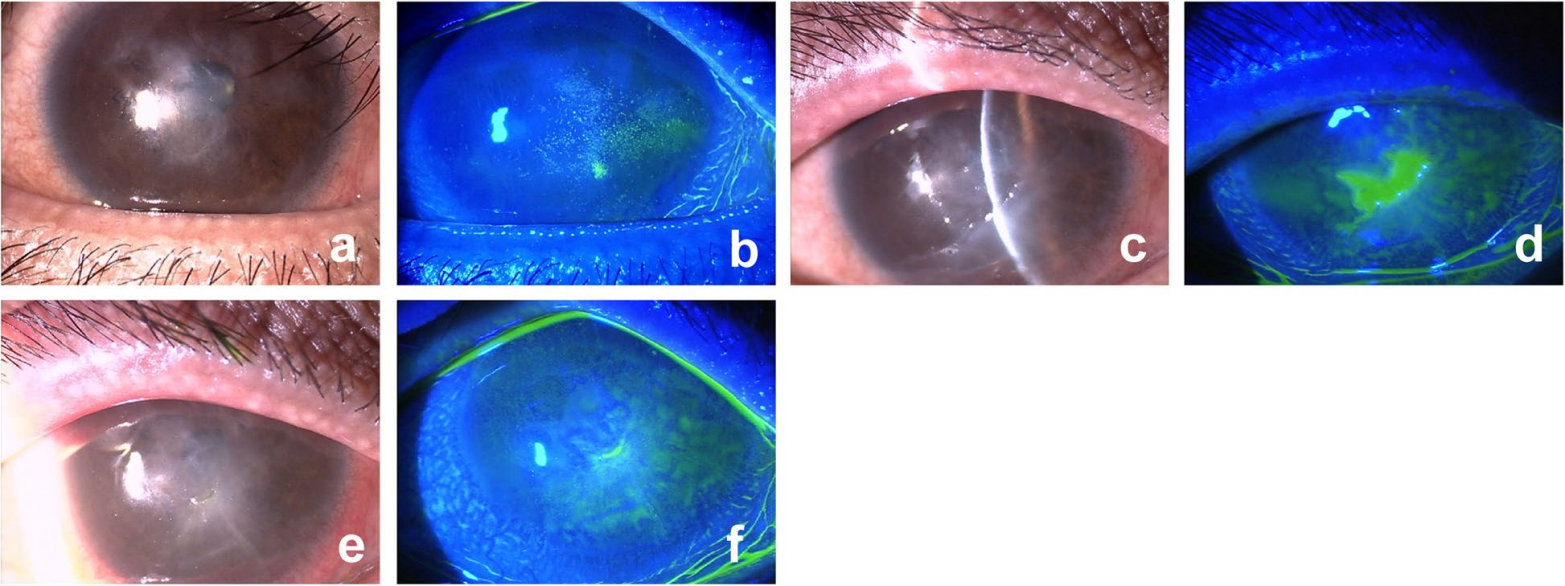
Clinical course and features of the patient with HSV keratitis. Slit-lamp examination photographs of a patient with HSV keratitis at Days 0, 42, and 49. **a**, and **b**: Photographs at Day 0 show corneal leucoma with fluorescein staining positive superficial punctate keratopathy. **c**, and **d**: Photographs at Day 42 show geographic keratitis with a dendritic tail. **e**, and **f**: Photographs at Day 49 show improvement in geographic keratitis. The area of epithelial defect with fluorescein staining gradually decreased. HSV, herpes simplex virus

A 45-year-old man (HSV2 in Table 1) with atopic keratoconjunctivitis (AKC) complicated by herpes simplex virus necrotizing keratitis in the right eye was followed up in the ophthalmology outpatient department of our hospital. AKC was active in his right eye, but necrotizing keratitis had healed into a corneal leukoma. After his initial visit two years ago, he presented to the outpatient department (Day 0) with a foreign body sensation in his right eye for several days. Bulbar conjunctival hyperemia, palpebral conjunctival hyperemia and swelling with papillary formation, and punctate corneal epitheliopathy were observed in his right eye (Figs. 3a and b). HSV DNA test using tear specimens of his right eye was performed using a microfluidic real-time PCR system; however, the results were under the lower detection limit. As a result, the ocular surface disease was diagnosed as AKC. His right eye with AKC with improved interstitial keratitis had been treated with acyclovir ophthalmic ointment (2 times per day), fluorometholone ophthalmic suspension 0.1% (one time per day), and moxifloxacin ophthalmic solution (2 times per day), and his left eye with AKC had been treated with tacrolimus ophthalmic suspension (2 times per day).

On Day 42, the foreign body sensation in his right eye recurred, and he revisited the outpatient department. Geographic keratitis was observed in his right cornea, and the result of HSV DNA test using tear specimens of his right eye was positive with 2.3 × 10^7^ copies/μL (Figs. 3c and d). The patient was treated with acyclovir ophthalmic ointment (5 times/day), fluorometholone ophthalmic suspension 0.1% (1 time/day), moxifloxacin hydrochloride (2 times/day), and valacyclovir 500 mg orally twice daily.

On Day 49, the geographic ulcer improved and HSV DNA value in tears was under the lower limit of detection (Figs. 3e and f). In this case, the microfluidic real-time PCR test was useful in the differential diagnosis between AKC and HSK, and in determining the efficacy of antiviral drugs against HSK. (Table 6).

**Table 6:** Clinical course and features of the representative patient with HSV keratitis

Time	Corneal findings	Topical treatment of eye drops	HSV DNA in tears (copies/μL)	
			**Right eye**	**Left eye**
Day 0	Interstitial keratitis (Healing stage)	**Right eye** Acyclovir ophthalmic ointment (2 times) Fluorometholone ophthalmic suspension 0.1% (1 time) Moxifloxacin ophthalmic solution (2 times) **Left eye** Tacrolimus ophthalmic suspension (2 times)	Negative	Negative
Day 42	Geographic keratitis (Recurrence)	**Right eye** Acyclovir ophthalmic ointment (3 times) Fluorometholone ophthalmic suspension 0.1% (1 time) Moxifloxacin ophthalmic solution (2 times) **Left eye** Tacrolimus ophthalmic suspension (2 times)	2.3 × 10^7^	Negative
Day 49	Corneal scar (Improved geographic keratitis)	Right eye Acyclovir ophthalmic ointment (1 time) Fluorometholone ophthalmic suspension 0.1% (1 time) Moxifloxacin ophthalmic solution (2 times) **Left eye** Tacrolimus ophthalmic suspension (2 times)	Negative	Negative

*HSV* herpes simplex virus

### Representative case of HZO

#### Case 1

**Fig. 4:**
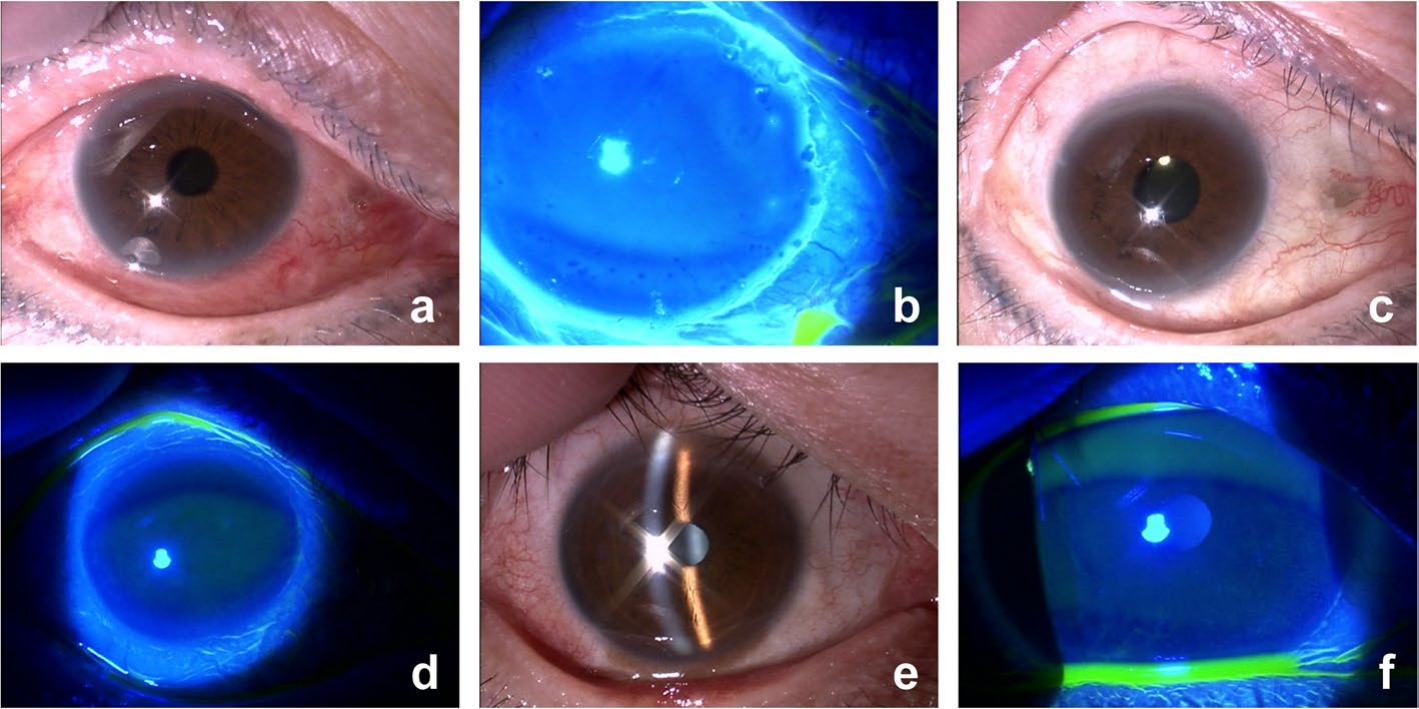
Clinical course and features of the patient with VZV keratitis. Slit-lamp examination photographs of the patients with varicella-zoster ophthalmicus. **a**, and **b**: Clinical findings of case 1 on Day 1 show multiple pseudodendritic keratitis with positive fluorescein staining and scleritis. **c**, and **d**: Clinical findings of case 1 on Day 22 show that varicella-zoster ophthalmicus improved. **e**, and **f**: Clinical findings of case 2 on Day 35 show improved keratitis and continued scleritis. VZV, varicella-zoster virus

A 74-year-old woman (VZV5 in Table 2) was referred to our ophthalmology department due to complaints of foreign body sensation and blurred vision in her right eye associated with herpes zoster infection in the area of distribution of the first branch of the trigeminal nerve. In the dermatological clinic one day earlier (Day 0), she was diagnosed with herpes zoster and was given antiviral medication. On Day 1, follicular conjunctivitis, pseudodendritic keratitis, and scleritis were observed during ophthalmological examination using a slit-lamp (Figs. 4a and b). During the first VZV DNA test on day 1, 3.9 × 10^6^ and 2.1 × 10^2^ copies/μL of VZV DNA were observed in right and left eyes, respectively, as shown in Table 7. She was treated with acyclovir systemically (750 mg/day of intravenous administration for 7 days), and acyclovir ophthalmic ointment instillation (5 times/day). The clinical course and features of the patient are shown in Figs. 4a and b. Finally, her keratitis and scleritis improved on Day 22 (Figs. 4c and d), and subsequently, the results of the VZV DNA test of both eyes were under the lower limit of detection.

**Table 7:** Clinical course and features of the representative patient with HZO: case 1

Time	Corneal findings	Topical treatment of eye drops	VZV DNA in tears (copies/μL)	
			**Right eye**	**Left eye**
Day 1	Severe pseudodendritic keratitis Scleritis	Acyclovir ophthalmic ointment (5 times)	3.9 × 10^6^	2.1 × 10^2^
Day 4	Mild pseudodendritic keratitis Scleritis	Acyclovir ophthalmic ointment (5 times) Betamethasone ophthalmic solution (2 times)	2.7 × 10^6^	Negative
Day 7	Punctate subepithelial keratitis Scleritis	Acyclovir ophthalmic ointment (3 times) Betamethasone ophthalmic solution (3 times)	4.0 × 10^5^	Negative
Day 15	Punctate subepithelial keratitis	Acyclovir ophthalmic ointment (3 times) Betamethasone ophthalmic solution (3 times)	NT	NT
Day 22	Corneal scar (Improved HZO)	Acyclovir ophthalmic ointment (2 times) Betamethasone ophthalmic solution (3 times)	Negative	NT

Abbreviations: *NT* not tested, *HZO* herpes zoster ophthalmicus, *VZV* varicella-zoster virus

#### Case 2

A 49-year-old man (VZV3 in Table 2) was referred to our ophthalmology department due to complaints of swelling and difficulty in opening the right eyelids, which was associated with herpes zoster infection in the area of distribution of the first branch of the trigeminal nerve.

In the dermatological clinic three days earlier (Day 0), he was diagnosed with herpes zoster and was given antiviral medication. On Day 3, follicular conjunctivitis, pseudodendritic keratitis, and scleritis were observed during ophthalmological examination using a slit-lamp. During the first VZV DNA test on day 1, 3.5 × 10^7^ and 9.9 × 10^3^ copies/μL of VZV DNA were observed in right and left eyes, respectively, as shown in Table 8. Subsequently, he was treated with famciclovir 1500 mg daily systemically, and acyclovir ophthalmic ointment instillation (5 times/day). On Day 31, his eyelid swelling and conjunctivitis improved; however, scleritis on the temporal side remained. In addition, the results of the VZV DNA test were under the lower detection limit on Days 7 and 31 in the left and right eyes, respectively. On Day 38, punctate subepithelial keratitis and scleritis (Figs. 4e and f) were observed in his right eye. Subsequently, the VZV DNA test result in his right eye was re-positive with 6.2 × 10^1^ copies/μL of VZV DNA. On Day 51, the punctate subepithelial keratitis and scleritis showed improvement in his right eye, and the VZV DNA test result was under the lower limit of detection.

**Table 8:** Clinical course and features of the representative patient with HZO: case 2

Time	Corneal findings	Topical treatment of eye drops	VZV DNA in tears (copies/μL)	
			**Right eye**	**Left eye**
Day 3	Punctate subepithelial keratitis scleritis	Acyclovir ophthalmic ointment 5 times	3.5 × 10^7^	9.9 × 10^3^
Day 10	Punctate subepithelial keratitis Scleritis	Acyclovir ophthalmic ointment 4 times Betamethasone phosphate sodium 2 times	7.4 × 10^5^	Negative
Day 17	Scleritis	Acyclovir ophthalmic ointment 2 times Betamethasone phosphate sodium 1 times	2.2 × 10^3^	NT
Day 31	Punctate subepithelial keratitis Scleritis	Acyclovir ophthalmic ointment 1 times Betamethasone phosphate sodium 1 times	Negative	NT
Day 38	Punctate subepithelial keratitis Scleritis	Acyclovir ophthalmic ointment 2 times Betamethasone phosphate sodium 2 times	6.2 × 10^1^	NT
Day 51	Scleritis	Acyclovir ophthalmic ointment 2 times Betamethasone phosphate sodium 2 times	Negative	NT

Abbreviations: *NT* not tested, *HZO* herpes zoster Ophthalmicus, *VZV* varicella-zoster virus

Monitoring of VZV DNA levels in tears of these patients indicated that acute phase HZO shows positive VZV DNA in tears, and careful follow-up is required in HZO patients with recurrent keratitis and scleritis due to re-elevated VSV DNA levels in tears.

## Discussion

We investigated the utility of the tear test for detecting HSV and VZV DNA in patients with herpetic ocular surface diseases as a clinical examination tool. In addition, in this study, herpetic keratitis including HSK and HZO was diagnosed using a microfluidic real-time PCR system. The current study demonstrated the following clinical advantages: 1) the time required for the detection of HSV and VZV DNA in tears with a microfluidic real-time PCR system was approximately 40 min; 2) the sensitivity and specificity of quantitative PCR test for HSV and VZV DNA in tears of the patients with infectious epithelial keratitis type of HSK and HZO were 100% each; 3) no cross-reaction between HSV and VZV DNA was observed.

Quantitative PCR for HSV DNA in tears is reportedly a useful diagnostic tool for HSK. Our previous multicenter study reported that the diagnosis of HSK using real-time PCR for HSV DNA had a sensitivity and specificity of 55.8% and 100%, respectively [5]. In particular, one of the factors that decreased the sensitivity of tear HSV DNA test in patients with HSK is the influence of the pre-test use of antiviral drugs such as acyclovir ophthalmic ointment, which reduces the number of HSV DNA copies. However, this study demonstrated no difference in sensitivity between the patients with acyclovir treatment and those without. In addition, background factors of herpetic ocular diseases that affect the amount of virus in tear fluid may include treatments with corticosteroids and immunosuppressive drugs, antibody immunotherapy, immune disorders such as collagen diseases and atopic dermatitis, and immunocompromised hosts. The influence of these background factors on the HSV/VZV DNA test in tears is an important issue for the future. Another factor that affects the sensitivity of the HSV DNA test is that the HSV DNA levels in tears vary depending on the clinical form and severity of HSK. The HSV DNA levels in tears are high in infectious epithelial keratitis including dendritic, geographic, and necrotizing keratitis, and low in disciform keratitis and endotheliitis [5, 10]. Therefore, it is important to set a suitable lower detection limit for the HSV DNA test to improve the sensitivity for the diagnosis of HSK. In the current study, the lower limit of detection of HSV DNA test was set at 7.6 × 10^1^ copies/μL; furthermore, the sensitivity and specificity of HSV DNA test were 100% each in patients with and without acyclovir treatment. These results may indicate the usefulness of this HSV DNA test as a clinical diagnostic tool for infectious epithelial HSK. In addition, a high copy number of HSV DNA in tears was detected in an HSK patient with refractory keratitis that resisted topical acyclovir treatment. We considered this a severe case in which the viral load was not sufficiently reduced by topical acyclovir treatment. Therefore, systemic administration of antiviral drugs should be considered for such refractive cases. Accordingly, a clinical test that can rapidly measure the number of HSV DNA copies in the tears of the patients being treated with antiviral drugs is a useful tool for determining the clinical treatment strategy.

In the patients with HZO, corneal findings including pseudodendritic keratitis, corneal subepithelial opacity, disciform keratitis, and interstitial keratitis were observed. These corneal lesions sometimes recur, resist treatment, and become refractory. For these patients with VZV keratitis, the copy number of VZV DNA in tears has been reported to be useful not only in diagnosis but also in determining the severity of the keratitis [11]. In the current study, the copy number of VZV DNA in tears of patients with acute HZO as well as the diagnostic rate (sensitivity) was high. In addition, acute phase HZO was positive for VZV DNA in the tears of both eyes, although the VZV DNA levels in the affected eyes were significantly higher than the unaffected ones. These results suggest that in the treatment of HZO, the need for topical ocular administration of antiviral drugs, in addition to systemic administration of antiviral drugs, should be considered, taking into account the viral load in tears. On the other hand, in our reported patients who were followed up by monitoring copy number of VZV DNA in tears, re-elevation of copy number of VZV DNA in tears was observed. This re-elevation of VZV in tears of patients with HZO has not been studied previously. Therefore, in patients with refractory HZO, VZV DNA levels in tears should be monitored and the results of tear testing should be incorporated into therapeutic drug selection, especially in the indication of steroids.

This study has some limitations. First, the current study did not evaluate the usefulness of HSV DNA test in tears in patients with herpetic stromal keratitis. Herpetic stromal keratitis is divided into disciform and necrotizing keratitis. The copy number of HSV DNA is high in patients with necrotizing keratitis and almost under the detection limit in disciform keratitis in HSV DNA tests in tears [5, 10]. Therefore, the copy number of HSV DNA in the tears of patients with herpetic stromal keratitis may help select an appropriate medical treatment. In other words, steroid eye drops, which are the main drugs used in the treatment of herpetic stromal keratitis, should be administered with caution to patients with high copy numbers of HSV DNA in tears. If we examine a patient with herpetic stromal keratitis, a rapid detectable test for HSV DNA in tears may be a crucial clinical test tool in routine clinical practice. The usefulness of this HSV DNA test in patients with herpetic stromal keratitis should be evaluated in large-scale studies in the future. Second, the usefulness of the HSV and VZV DNA tests for patients with concomitant herpetic endotheliitis and uveitis is unclear because the present patient group did not include any cases of corneal endotheliitis and uveitis. HSV and VZV DNA testing of the aqueous humor is useful in diagnosing herpetic corneal endotheliitis; however, the usefulness of the tear test should be accurately verified. In addition, examining the usefulness of this test system for detecting HSV DNA and VZV DNA in aqueous humor may provide more certainty in the differential diagnosis of corneal endotheliitis and infectious uveitis. Third, viral DNA presumed to be shedding has been detected in the unaffected eyes of both HSK and HZO patients. The establishment of criteria to determine whether the amount of detected viral DNA has pathological significance or not is an important issue in this assay.

## Conclusions

Rapid quantitative PCR for HSV and VZV DNA in tears using a microfluidic real-time PCR system is useful for diagnosing, appropriate use of therapeutic agents, and monitoring HSK and HZO.

## Data Availability

The data that support the findings of this study are available from the corresponding author, S.Y., upon reasonable request.

## Abbreviations

AKC: Atopic keratoconjunctivitis
HHV: Human herpes virus
HSV: Herpes simplex virus
HSK: Herpes simplex keratitis
PCR: Polymerase chain reaction
HZO: Herpes zoster ophthalmicus
VZV: Varicella-zoster virus
